# Late Gadolinium Enhancement by Cardiac Magnetic Resonance and Speckle Tracking Echocardiography in the Evaluation of Cardiac Complications in Chagas Cardiomyopathy: A Systematic Review

**DOI:** 10.31083/j.rcm2309323

**Published:** 2022-09-16

**Authors:** Laura-M Romero Acero, Andrés-D Gallego Ardila, Michele Nanna, Frida T Manrique Espinel, Héctor M Medina, Esteban Sciarresi, Fabio-A Tabares-Mora, Alejandro Olaya Sanchez, Carolina Ayala, Jorge L Fajardo Ruge, Ramón Medina-Mur, Diana Vargas Vergara, Gabriel Salazar Castro, Andrés Díaz

**Affiliations:** ^1^Cardiology Department, Hospital de San José, Fundación Universitaria de Ciencias de la Salud-FUCS, 110321 Bogota, Colombia; ^2^Research Department, Hospital de San José, Fundación Universitaria de Ciencias de la Salud-FUCS, 110321 Bogota, Colombia; ^3^Cardiology Department, Albert Einstein Hospital, Albert Einstein College of Medicine, Bronx, NY 10461, USA; ^4^Non-invasive methods Department, Universidad del Rosario, La Cardio-Fundación Cardio-infantil, 110131 Bogota, Colombia; ^5^Cardiovascular Diagnostic Imaging Department, Universidad del Rosario, La Cardio-Fundación Cardio-infantil, 110131 Bogota, Colombia; ^6^Cardiology Department, Universidad Abierta Interamericana Rosario, Instituto de Cardiología de San Nicolás, 2900 San Nicolás De Los Arroyos, Buenos Aires, Argentina; ^7^Cardiology Department, Universidad del Bosque, La Cardio-Fundación Cardio-infantil, 110131 Bogota, Colombia

**Keywords:** Chagas disease, Chagas cardiomyopathy, magnetic resonance imaging, speckle tracking echocardiography

## Abstract

**Background:**

Chagas cardiomyopathy (CC) increases cardiovascular mortality associated with congestive heart failure (CHF), ventricular arrhythmias (VA), and sudden cardiac death (SCD). Different imaging techniques have been tested to assess disease progression and cardiac risk in individuals with Chagas disease (ChD). In this systematic review, we evaluated the accuracy in detecting cardiac complications in CC patients using cardiac magnetic resonance (CMR) and speckle tracking echocardiography (STE).

**Methods:**

A search was done on PubMed, Cochrane, and Embase for studies in humans over 18 years of age with ChD. Demographic data, research methodology, imaging parameters, and cardiac outcomes were extracted, and study quality was assessed, resulting in a narrative description.

**Results:**

Twelve studies with 1124 patients were analyzed. One study discovered a contractility pattern by STE. Four studies assessed the identification of Early Cardiac Impairment (ECI) and VA risk, respectively, while three studies evaluated the risk of SCD. Global Longitudinal Strain (GLS) identified patients with ECI (–18.5 ± 3.4% non-fibrosis vs –14.0 ± 5.8% fibrosis, *p* = 0.006 and –18 ± 2% non-fibrosis vs –15 ± 2% fibrosis, *p* = 0.004). The amount of fibrosis >11.78% or in two or more contiguous transmural segments were markers for VA risk. GLS and the amount of fibrosis were found to be predictors of SCD.

**Conclusions:**

STE may be considered a screening technique for identifying the subclinical status of CHF. CMR using Late Gadolinium Enhancement (LGE) is considered a relevant parameter for stratifying patients with ChD who are at risk of SCD. Fibrosis and GLS can be used as markers to categorize patients at risk for arrhythmias.

## 1. Introduction 

Chagas disease (ChD) is an infectious illness that is a public health problem in Latin America. Globalization has increased the number of cases to approximately 300.000 in the United States and 181.181 in Europe [1]. ChD is defined by an acute phase that progresses to the indeterminate form (IF) [2], which in a minority of patients evolves to the clinically active determined form. ChD can cause chronic myocyte inflammation and fibrosis in the clinically active form, resulting in Chagas cardiomyopathy (CC), which can cause congestive heart failure (CHF), sudden cardiac death (SCD), and ventricular arrhythmias (VA). CHF accounts for more than 50% of mortality in ChD, and SCD is a relatively common feature of the disease [3]. In comparison to other kinds of cardiomyopathies, CC is associated with a lower rate of survival [4].

Despite significant research efforts in ChD, robust markers or indicators for guiding therapeutic interventions such as implantable cardioverter-defibrillators destined to treat lethal arrhythmias and abort episodes of SCD, have not been established.

As a result, early diagnosis of cardiac abnormalities leading to overt disease has become a major research priority with a potential impact on long-term prognosis. Different non-invasive diagnostic methods such as cardiac magnetic resonance (CMR) using Late Gadolinium Enhancement (LGE) and speckle tracking echocardiography (STE) have been used to identify CC. CMR is considered the goldstandard to assess ventricular systolic function, detect segmental wall motion abnormalities, and measure the amount of fibrosis in the myocardium by LGE assessment [4, 5]. STE also provides vital insights into myocardial dynamics and the mechanical function of the heart [2].

However, there is no consensus on which imaging modality is the best choice based on the cardiac complication being assessed in CC. Nor is there any consensus on whether LGE or STE is better for detecting an IF of ChD. To help classify risk and create effective early interventions to avoid or minimize the most severe cardiac outcomes associated with CC, specific criteria related to each method must be established. This article will review and synthesize the information about the accuracy of CMR and STE in detecting cardiac complications in CC patients.

## 2. Methods 

This systematic review of the literature compared the accuracy of two imaging techniques CMR and STE in for detecting cardiac complications in CC. The work was published in PROSPERO under the ID CRD42021272533, utilizing Preferred Reporting Items for Systematic Reviews and Meta-Analyses (PRISMA).

Pubmed, Embase, and Cochrane were searched electronically using English, Spanish and Portuguese languages. The search terms for STE databases were: “Speckle tracking echocardiogram”, “Speckle tracking strain”, and “Myocardial strain”. “Magnetic Resonance Imaging” was used as a key term for LGE. Finally, the terms for ChD were: “Chagas Cardiomyopathy” and “Chagas disease”. Search equations also included Boolean terms (AND/OR) (Appendix Table 2). Additionally, a Google Scholar search was done to locate unpublished literature or research not available in the electronic databases. References from systematic reviews obtained during the search were screened and experts recommended adding additional articles.

Two researchers chose which publications to include (screening was done by title, abstract, and secondly, full text revision). Inclusion criteria for articles were defined as: studies done on humans over 18 years old, patients with ChD, experimental or observational studies and studies that included speckle tracking and/or LGE techniques. Exclusion criteria included: low quality assessment and case reports or series of cases. A third reviewer (epidemiologist) was consulted in the event of a dispute.

After completing the selection procedure, the study team extracted data using a form which was designed, tested, and standardized. The extraction matrix has six sections: (1) first author’s last name, publication year, journal, DOI, country, study design, period of the study, and number of groups; (2) imaging methods and equipment; (3) participants’ age, gender, and race; (4) CMR variables included: left ventricle contractility and location, fibrosis presence and location in T1 [enhancement], left ventricular ejection fraction (LVEF); (5) echocardiography variables included ventricular dilatation (global or segmental), wall motion abnormalities (global or segmental), LVEF; longitudinal, radial, and circumferential strains (global and segmental); (6) cardiac outcomes: SCD, VA, and CHF. Finally, valvulopathy was excluded since Trypanosoma.cruzi does not directly affect heart valves.

Quality of information was assessed by Joanna Briggs Institute (JBI) check list forms based on each study design. The epidemiologist applied the tool to the different articles and categorized the quality of information using three levels: high, moderate, and low (Table 1 (Ref. [3, 5, 6, 7, 8, 9, 10, 11, 12, 13, 14, 15])/Appendix Table 3 (Ref. [3, 5, 6, 7, 8, 9, 10, 11, 12, 13, 14, 15])). For data synthesis and analysis, a narrative description was built that included characteristics and differences found in primary studies for each imaging technique (STE and LGE) with respect to the cardiac outcomes defined. A consensus of imaging technique experts was performed to unify recommendations for cardiac complications in patients with ChD identified throughout the literature review.

**Table 1.** **Results from the systematic review. Summary of socio-demographic characteristics, type of imaging techniques, the main outcomes of the systematic review and quality assessment**.

Last name of the first author	N	Country of publication	Age (mean ± SD) or (mean/range)	Imaging technique	Gender	Measurement of CI		Outcomes		
						Value	Cutoff	CHF	AR	SCD
${\mathrm{BARROS}}^{1}$ 2015 [3]	62	Brazil	58.3 ± 8.3	STE	Male (62%)	GLS	–14.3%	None	Predictor for malignant VAR	None
${\mathrm{MELENDEZ}}^{1}$ 2019 [9]	54	Mexico	55.9 ± 12.2	LGE	Male (57%)	Percentage of fibrosis	>17.1%	None	Predictors For V-AR	None
						2 or more contiguous, transmural segments with fibrosis	None			
${\mathrm{MELLO}}^{2}$ 2012 [6]	50	Brazil	55.1 ± 11.9	LGE	Male (72%)	2 or more contiguous, transmural segments with fibrosis	None	None	Predictor for V-AR (4.1 fold greater risk)	None
${\mathrm{TASSI}}^{2}$ 2014 [5]	61	Brazil	62.32 ± 10.43	LGE	Male (38%)	Percentage of fibrosis	>11.78%	None	Predictor for V-AR	None
${\mathrm{NOYA-RABELO}}^{2}$ 2018 [7]	61	Brazil	58 ± 8	LGE	Male (41%)	Percentage of fibrosis indeterminate form group (41%) vs cardiac form without lv dysfunction group (44%)	None	Early detection of subclinical	None	None
								CHF stage		
${\mathrm{VOLPE}}^{1}$ 2018 [12]	140	Brazil	57/(45–67)	LGE	Male (48%)	Amount of fibrosis (9.2% median calculated among patients with scar)	None	None	Predictor for sustained ventricular tachycardia	Predictor of sudden cardiac death
${\mathrm{LIMA}}^{2}$ 2015 [13]	131	Brazil	55 ± 10	STE	Male (34%)	RD (mm) pattern of contraction - CCC suspected	None	None	None	None
						Inferior segment Ch3 0.92 ± 1.72 mm vs C3 2.22 ± 1.20 mm, *p* = 0.03				
						Posterior segment Ch3 2.02 ± 0.90 mm vs C3 3.80 ± 1.92 mm, *p* = 0.03				
						Septal segment Ch3 5.88 ± 2.25 mm vs C3 2.39 ± 1.09 mm, *p* = 0.001				
						Anterior segment Ch3 5.27 ± 2.49 mm vs C3 3.62 ± 1.50 mm, *p* = 0.04				
${\mathrm{CIANCIULLI}}^{2}$ 2020 [14]	90	Argentina	59/(52–65)	STE	Male (40%)	LV-GLS	–18%	Early detection of subclinical CHF stage	None	None
						LV-GLS by segments (%)	Basal anteroseptal: ChD –17 (–14–19) vs CG 19 (–17–21) *p* = 0.09			
							Mid-anterior: ChD –15 (–12–18) vs CG 20 (–19–22) *p* = 0.002			
							Apical-anterior: ChD –16 (–10–21) CG –22 (–18–25) *p *< 0.001			
							Mid-lateral: ChD –17 (–13–20) vs CG –19 (–18–22) *p* = 0.16			
							Mid-posterior: ChD –17 (–14–20) vs CG20 (–17–22) *p* = 0.38			
							Basal inferoseptal: ChD –17 (–14–20) vs CG –18 (–16–14) *p* = 0.99			
							Apex: ChD –17 (–12–21) vs CG –22 (20–25) *p *< 0.001			
${\mathrm{ROMANO}}^{2}$ 2020 [8]	65	Brazil	52 ± 11.3	STE/LGE	Male (50%)	LV-GLS	–18.5 ± 3.4% (Absence of fibrosis) *p* = 0.006 –14.0 ± 5.8 (Presence of fibrosis) *p* = 0.0006	Early detection of subclinical CHF stage	None	None
						LV-GLS by segments (%)	Basal inferoseptal:			
							NC –15.2 ± –2.7			
							IFCD –13.1 ± 3.4			
							CCC –11.9 ± 5.6 *p* = 0.01			
							Basal inferior:			
							NC –18 ± –2.2			
							IFCD –16.3 ± 3.3			
							CCC –11.9 ± 5.6 *p* = 0.01			
							Mid inferoseptal:			
							NC –19.4 ± 2.0			
							IFCD –17.7 ± –3.2			
							CCC –14.5 ± 6.3 *p* = 0.03			
							Mid inferolateral:			
							NC –17.8 ± 2.8			
							IFCD –15.2 ± 3.5			
							CCC –10.5 ± 8.2 *p* = 0.01			
${\mathrm{GOMES}}^{2}$ 2016 [15]	168	Brazil	45 ± 8	STE/LGE	Male (45%)	GLS (%)	–18.5 ± 1.8 (NF) vs –15.0 ± 1.8 (F) *p* = 0.004	Early detection of subclinical CHF stage	None	None
						GCS (%)	–18.6 ± 2.4 (NF) vs –13.8 ± 2.2 (F) *p* = 0.002			
						GRS (%)	54 ± 12 (NF) vs 36 ± 13 (F) *p* = 0.02			
SANTOS ${\mathrm{JUNIOR}}^{2}$ 2019 [11]	112	Brazil	56.7 ± 11.8	STE	Male (56%)	GLS (%)	>–12%	Greater risk for hospitalization and heart transplant	None	Greater risk
${\mathrm{SENRA}}^{2}$ 2018 [10]	130	Brazil	53.6 ± 11.5	LGE	Male (46.1%)	Amount of fibrosis	≥12.3G	None	Higher risk	Higher risk

N, Number of patients who participated in the study; CI, Cardiac Impairment; CHF, Congestive Heart Failure; AR, Arrhythmia; SCD, Sudden Cardiac Death; GLS, Global Longitudinal Strain; V-AR, Ventricular Arrhythmia; RD, Radial Displacement; Ch, Chagas Group; C, Control Group; LV-GLS, Left Ventricular Global Longitudinal Strain; G, Grams; STE, Speckle Tracking Echocardiography; LGE, Late Gadolinium Enhancement; NC, Control Group. IFCD, Indeterminate Form Chagas group; CCC, Chagas Cardiomyopathy Group; GCS, Global Circumferential Strain; GRS, Global Radial Strain; NF, No Fibrosis Group; F, Fibrosis Group; SD, Standard Deviation; Quality assessment: ${\mathrm{High}}^{1}$; ${\mathrm{Moderate}}^{2}$.

This manuscript adheres to the ethical principles of health research. During the selection process of the articles included and the data analysis, the veracity of the information has been rigorously handled.

## 3. Results

**Fig. 1.**
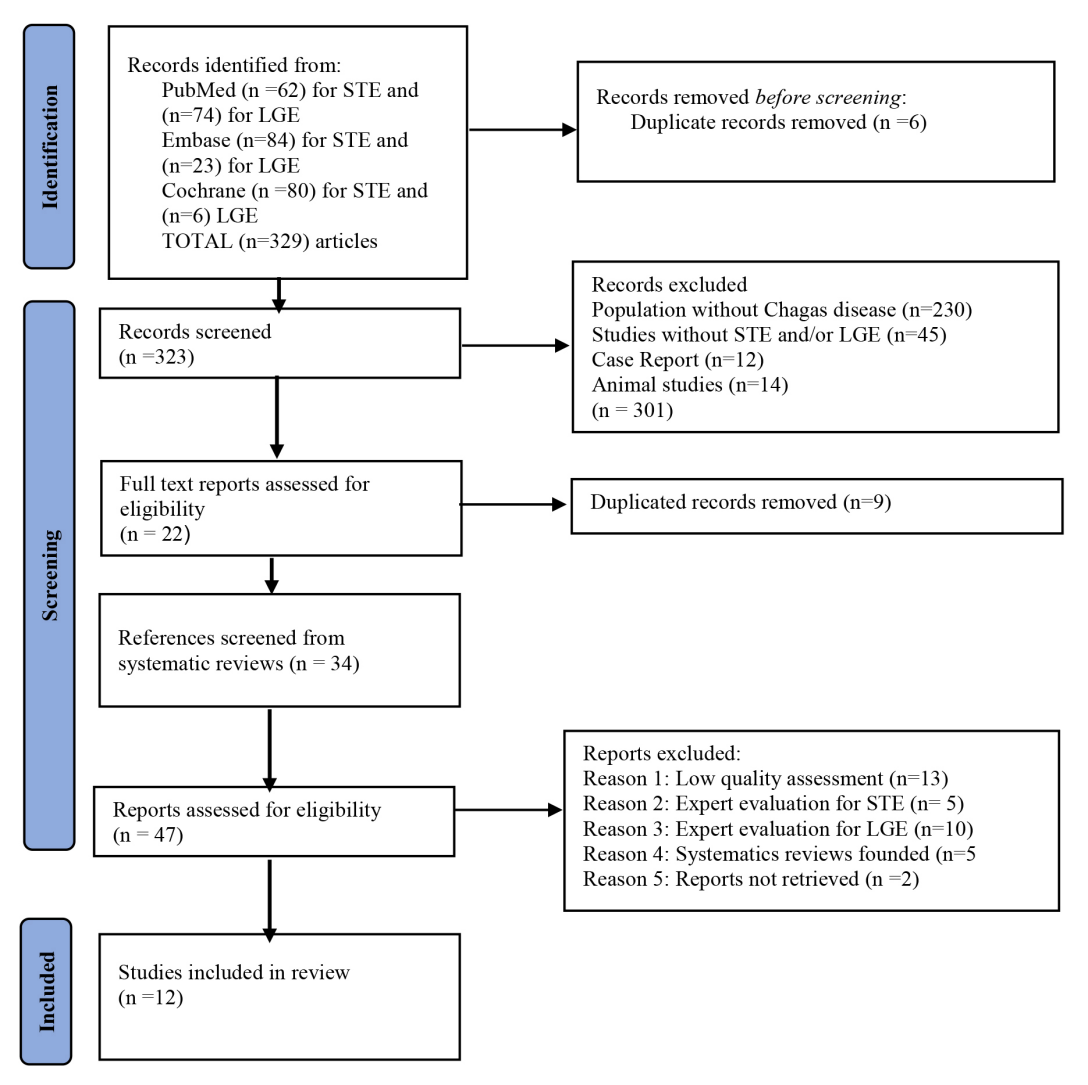
**PRISMA, systematic research flow diagram**.

Through database searches, 329 studies were located. Six duplicate records were removed resulting in a total of 323 articles. These articles identified 22 studies that met the criteria for full-text evaluation. After reviewing these 22 documents for inclusion and exclusion criteria, nine articles were eliminated as duplicates. Five systematic reviews (SRs) were identified in the full text reports, and the SRs included 34 references. As a result, 47 articles were evaluated: thirteen were excluded due to low quality assessment, fifteen were excluded based on expert opinion evaluation, five were excluded since they were identified as SRs, and two were not recovered. Finally, 12 articles were included in the study. The method for performing this systematic review is depicted in Fig. 1.

The flow diagram depicts the different phases developed to obtain the selected articles. It lists the studies identified, included and excluded, and the reasons for exclusions. PRISMA: Preferred Reporting Items for Systematic Reviews and Meta-Analyses.

Six of the twelve studies [3, 5, 6, 7, 8, 9] were cross-sectional, two were cohorts (one was retrospective and one was prospective) [10, 11], and four were case-control [12, 13, 14, 15]. Most studies are categorized as high to moderate quality (Table 1/Appendix Table 3). The studies included 1124 patients from Brazil, Mexico, and Argentina (946 ChD and 178 non-ChD). They ranged in age from 45 to 62 years old [5, 15], and most participants were male (590 = 52.4%) [3, 6, 8, 9, 10, 11, 13, 15].

In terms of imaging techniques, six used LGE via CMR [5, 6, 7, 9, 10, 12], two LGE/STE [8, 15], and four STE [3, 11, 13, 14]. A modified Second Brazilian Consensus on ChD was acknowledged in each paper. The studies analyzed cardiac outcomes in patients with ChD such as: CHF, VA, and SCD. Table 1 highlighted the general characteristics of articles considered and summarizes key sociodemographic and cardiac outcome data while the graphical abstract summarized imaging recommendations to assess cardiac complications in patients who have ChD.

## 4. Cardiac Outcomes as Measured by STE and LGE 

CC subclinical phase (IF) was evaluated across several studies utilizing global and segmental strains. Cianciulli *et al*. [14] demonstrated that the ChD group had a reduced left ventricular (LV)-global longitudinal strain (LV-GLS) in comparison to the control group (median –17 vs –20.3, *p *< 0.001). Seven segments were significantly lower –18% in patients with ChD compared to the control group: mid-anterior –15% (–12 to –18) vs –20% (–19 to –22), apical- anterior –16% (–10 to –21) vs –22% (–18 to –25), and the apex –17% (–12 to –21) vs –22% (–20 to –25).

Similarly, Romano *et al*. [8] revealed that the indeterminate group’s regional longitudinal segments strain (RegLS) values were greater (worse) than those of the control group (NC): basal inferior-septal (–13.1 ± 3.4 vs –15.2 ± 2.7), basal-inferior (–16.3 ± 3.3 vs –18.6 ± 2.2), mid infero-septal (–17.7 ± 3.2 vs 19.4 ± 2.0), mid infero-lateral (–15.2 ± 3.5 vs –17.8 ± 2.8). The IF group had a lower global radial strain (GRS) than the NC group (28.4 ± 14.6 vs 34.2 ± 10.3; *p* = 0.043), and the CC group had a lower global longitudinal strain and global circumferential strain (GCS) than the NC group (GLS: –14 ± 6.3% vs –19.3 ± 1.6%, *p* = 0.001; GCS: –13.6 ± 5.2% vs –17.3 ± 2.8%; *p* = 0.008). Patients without fibrosis had significantly higher values of GLS (–18.5 ± 3.4 vs –14.0 ± 5.8, *p* = 0.006) compared those with detected fibrosis. Furthermore, a statistically significant non-linear relationship between GLS and fibrosis was observed in patients with ChD (r = 0.625, *p *< 0.001).

Gomes *et al*. [15] discovered that individuals with fibrosis in the early stages of the cardiac form had a reduced global strain value: GLS (–15 ± 2% vs –18 ± 2%, *p* = 0.004), GCS (–14 ± 2% vs –19 ± 2%, *p* = 0.002), and lower radial left ventricular strain (36 ± 13% vs 54 ± 12%, *p* = 0.02).

Lima *et al*. [13] described a peculiar imaging distribution pattern of contractility characteristic of CC. This study utilized the myocardial strain to assess left ventricular mechanics in four groups of patients with ChD and various progressive degrees of LV dysfunction from normal to severe (Ch1A, Ch1B, Ch2, and Ch3) compared to the control groups with matched degrees of LV dysfunction (C1, C2, C3). ChD groups had lower global longitudinal velocity values than control groups. However, a vicarious pattern was noted in the severe LV dysfunction ChD (Ch3) compared to a control group (C3): Ch3 displayed a surprising rise in global longitudinal and radial displacement (RD). In RD, segmental measurements revealed that Ch3 had lower values in the inferior and lateral wall segments vs C3 (RD posterior: Ch3 2.02 ± 0.90 mm vs C3 3.80 ± 1.92 mm, *p* = 0.03; inferior: Ch3 0.92 ± 1.72 mm vs C3 2.22 ± 1.20 mm, *p* = 0.03) and higher values in the septal and anterior wall segments vs C3 (RD antero-septal: Ch3 5.88 ± 2.25 mm vs C3 2.39 ± 1.09 mm, *p* = 0.001; anterior: Ch3 5.27 ± 2.49 mm vs C3 3.62 ± 1.50 mm, *p* = 0.04).

## 5. Myocardial Fibrosis and GLS 

Fibrosis was also used to evaluate the IF of ChD. A cross-sectional study comparing individuals with indeterminate ChD to those with the CC but no left ventricular dysfunction found that fibrosis occurred equally in individuals with indeterminate ChD (6/17; 41% of fibrosis) and individuals with the CC but no left ventricular dysfunction (7/16; 44% of fibrosis; *p* = 1.0). When the total quantity of myocardial fibrosis was compared between groups, it was determined to be 4.1% (IIQ: 2.1–10.7) in the indeterminate group, 2.3% (IIQ: 1–5) in the CC without ventricular dysfunction group, and 15.2% in the ventricular dysfunction group (IIQ: 1–5) [7].

GLS and cardiac fibrosis were both associated with an increased risk for cardiac events. Santo Junior *et al*. [11] demonstrated that GLS was an independent predictor of cardiac adverse events (HR 1.365; 95% CI 1.106–1.686; *p* = 0.004) with a value greater than >–12% thus increasing the likelihood of cardiac events (log-rank *p* = 0.035). Senra *et al*. [10] defined a cutoff value of ≥12.3 g for cardiac fibrosis that was found to be a good predictor of both the combined endpoint (adjusted HR 1.031; 95% CI 1.013–1.049; *p* = 0.001) and all causes of mortality (adjusted HR 1.028; 95% CI 1.005–1.051; *p* = 0.017). Each additional gram of myocardial fibrosis was related to a 2.8% increase in mortality and a 3.1% increase in the risk of reaching a combination of hard endpoints.

Volpe *et al*. [12] demonstrated that the scar (ascertained by LGE) group was more likely to experience a cardiac event related to primary (log-rank test, *p* = 0.043) and secondary (log-rank test, *p* = 0.016) endpoints, but there was no relationship between primary and secondary endpoint for the scar pattern. As a consequence, scars in patients with ChD is a strong predictor of sustained ventricular tachycardia and all causes of death.

## 6. Ventricular Arrhythmias 

VA were investigated in three studies involving LGE. Two studies established that the involvement of two or more transmural segments was associated with a higher risk of ventricular tachycardia (VT) [6, 9]. Mello *et al*. [6] found that the group of patients with VT had a relative 4.1 risk (95% CI 1.06–15.68) of having two or more segments with LGE with a transmural distribution, compared to those without VT. In addition, Melendez *et al*. [9] established a cutoff of 17.1% for cardiac fibrosis with respect to the percentage of LGE, resulting in 0.5 g (0–2.5) in the indeterminate group, 12 g (0.38–22) in the CC group without VT, and 23 g (18–34) in the CC group with VT. Tassi *et al*. [5] showed evidence that the presence of VA was associated with a cutoff point of >11.78% for cardiac fibrosis mass (*p *< 0.001) by LGE. LVEF and fibrosis were found to be inversely proportional ($R^{2}$ = –0.37).

Similarly, GLS was found to be associated with malignant VA in patients with CC. GLS was considerably worse in patients with arrhythmias than in the control group in a cross-sectional study (–13.6 ± 5.5 vs –16.5 ± 4.3, *p* = 022). GLS and LVEF all demonstrated the ability to differentiate between patients who did not have or had previous implantable cardioverter-defibrillators. A GLS cutoff of –14.3% sensitivity of 67% and a specificity of 69% for detecting VA, were found to be independent predictors of malignant arrhythmias [3].

## 7. Discussion 

The current review analyzed relevant literature pertaining to the ability of STE and LGE to detect CC complications—including their accuracy in predicting SCD, VA, and CHF. As a result, this systematic review gathered specific imaging findings to guide clinicians in the management of cardiac complications in patients with CC.

The only follow-up on the majority of patients in the IF of ChD is an electrocardiogram to assess progression to the cardiac form of the disease. Frequent sophisticated cardiac imaging is still not advised in asymptomatic patients for the assessment of heart disease [16]. Nonetheless, STE has the potential to provide detailed analysis of ventricular mechanics and myocardial contraction while allowing for the detection of subtle changes in the heart muscle prior to the beginning of cardiac complications [13]. Because STE can detect slight alterations in the fibers of the heart, it is beneficial for assessing the subclinical status of CHF in CC. Cianciulli *et al*. [14] concluded that GLS and segmental longitudinal peak systolic strain (SLPSs) values were sensitive enough to detect cardiac impairment in IF of ChD patients.

Romano *et al*. [8] also demonstrated that RegLS was greatly reduced in the IF population despite the absence of detectable myocardial fibrosis with LGE indicating that, in patients with IF ChD, the mere presence of RegLS abnormalities even in absence of myocardial fibrosis is a prelude to myocardial dysfunction.

In regard to fibrosis, Noya Rabelo *et al*. [7] demonstrated that myocardial fibrosis is not a good predictor of IF progression to an overt cardiac form. However, the degree of fibrosis is an excellent marker of CHF progression and severity of disease in individuals with CC. Fibrosis has been proven to be inversely related to LVEF. This implies that a greater level of fibrosis results in lower ventricular systolic function [5] and, that the extent of fibrosis correlates well with the New York Heart Association functional class [17]. The reduced myocardial contractility associated with fibrosis translates into reduced GLS. Romano *et al*. [8] found that GLS was modified to a greater extent when the quantity of fibrosis was larger. Gomes *et al*. [15] demonstrated that 50% of participants in stage A of ChD had lower GLS and GCS compared to the amount of cardiac fibrosis detected by CMR at follow-up, whereas those with normal GLS and GCS did not develop cardiac fibrosis at the follow up CMR examination. This observation has obvious relevance in a clinical setting since the onset of fibrosis in CC causes extensive ventricular remodeling and results in an increased likelihood of lethal arrhythmias and adverse outcome events [16].

Although, poor prognosis in CC may be driven by malignant VA and, consequently SCD, there is strong evidence that progressive CHF has a bigger impact as the most common cause of death in ChD [18]. As a result, identifying patients with early cardiac impairment by STE could be a key imaging technique to change prognosis in patients with ChD [8, 14, 15].

Because CC is considered arrhythmogenic, identifying people who are at risk of developing VA is critical. Myerburg *et al*. [19] considered myocardial fibrosis to be the arrhythmogenic substrate of VA. As a result, fibrosis has emerged as an important predictor of VA in ChD [5, 16]. Although LV dysfunction is an equally important substantial predictor of arrhythmic mortality in ChD, LVEF alone does not predict VA [20, 21, 22]. The Tassi, Mello, and Melendez studies indicated that the extent of cardiac fibrosis is a marker for VA (Table 1) [5, 6, 9]. Accordingly, correlating LV dysfunction with the amount of fibrosis could be extremely useful for clinicians in categorizing high risk arrhythmogenic patients.

STE does not detect fibrosis, but, by detecting functional abnormalities associated with fibrosis might help in risk stratification. For example, STE, by detecting the presence of abnormalities in the inferolateral and inferoseptal segments, common sites of origin for ventricular tachycardia in CC [23] helps in identifying patients at risk for malignant arrhythmic events, regardless of LVEF [3].

Considering LGE is a reliable quantification method for the amount of fibrosis and STE is a detector of variations in the segmental function of the myocardium, it is reasonable to state that for an evaluation of VA in ChD, patients should be evaluated by both techniques although this will depend on the availability of each technique and the expertise of physicians.

Predicting SCD in patients with ChD is difficult since it can be the first manifestation without previous symptoms or not be related to any other condition other than ChD [24]. The fibrotic nature of ChD makes arrhythmogenic events more likely, and these are the major cause of sudden death in patients with ChD [3].

LGE is excellent for quantifying the amount of myocardial fibrosis that is a predictor of SCD, and it has a clear role in stratifying mortality risk in patients with ChD [16]. Volpe *et al*. [12] demonstrated that LGE is an independent predictor of the composite endpoint of cardiovascular death and sustained ventricular tachycardia. Furthermore, Senra *et al*. [10] discovered that every additional gram of fibrosis increased the likelihood of hi developing major adverse cardiovascular events such as the risk of all causes of mortality, regardless of heart disease status (CHF, ventricular dysfunction, and arrhythmias). This finding provides support for the belief that fibrosis, apart from LV systolic function, is a useful indicator of cardiovascular adverse outcomes. Indeed, the American Heart Association has advised using CMR on patients with CC who have been diagnosed with complex ventricular arrhythmias [16].

Myocardial fibrosis has been shown to be a modifier of GLS as evidenced by the Romanos and Gomes studies, in which GLS decreased in the presence of fibrosis [8, 15]. This observation is important in stratifying the risk to SCD patients because it implies that, just as the degree of fibrosis is recognized as a predictor of SCD, GLS could be another predictor of mortality in CC. Santos-Junior *et al*. [11] noticed an association between GLS >–12% and the incidence of cardiovascular events, regardless of LV function. Based on data, using both approaches broadly and evaluating GLS as a predictor of mortality in CC is proposed.

Comparing CC to ischemic cardiomyopathy reveals that LVEF is a strong predictor of mortality in patients with heart disease [25, 26]. However, GLS in CC is more accurate than LVEF in quantifying ventricular function and predicting VA risk [3, 5]. Similarly, the amount of fibrosis is a good predictor of SCD in ChD, but LVEF is not [10]. As a result, adverse cardiac predictors in CC differ from other types of cardiomyopathies.

Finding imaging patterns to distinguish between specific types of cardiomyopathies could have a significant impact on the health system and make diagnosis easier. LGE has a long history of finding patterns in imaging to diagnose various cardiomyopathies [27]. However, Lima *et al*. [13] demonstrated that STE may also be utilized for the same purpose. Thus, when CC is suspected, there is a STE heterogenous pattern in the inferior and posterior walls of the LV as well as the septal and anterior regions that can be used to distinguish CC from other forms of cardiomyopathies.

## 8. Study Limitations 

Since the studies included in this systematic review were observational, there is a greater likelihood of increasing the risk of bias as a result of the lack of randomized clinical trials. Therefore, multi-center clinical trials are needed to validate the use of these two imaging techniques for the evaluation of cardiac complication of ChD. Due to the heterogeneity of the methodology studies and the data reports, a metaanalysis could not be performed.

STE and CMR using LGE should be interpreted by qualified observers. Although almost all studies indicated that inter- and intra-observer variability was controlled, there are no international recommendations that standardize CC measurement parameters.

This investigation limited its analysis to CMR using LGE. Other magnetic resonance imaging techniques such as T2 weighted sequence and T1 weighted myocardial early gadolinium enhancement sequence have been used in patients with ChD and reflect different stages of the natural history of the disease characterized by edema and hyperemia respectively. These are promising emerging tools, however, they have not been compared directly with STE and thus, they have been excluded from this analysis [28].

Surprisingly, this systematic review contained no papers from beyond Latin America. While ChD is endemic in this region, it is vital to bring it to the global community’s attention because CC is becoming increasingly common in non-endemic areas and the index of suspicion should be kept high even in nonendemic areas.

## 9. Conclusions

STE and LGE can be utilized to evaluate cardiac complications in patients with CC. Progression from IF to cardiac form can be assessed with STE because it is a tool that is sensitive enough to assess the subclinical status of CHF in ChD. But when the CC is established, the progression to CHF should be evaluated by correlating systolic ventricular function with the amount of fibrosis and GLS in order to predict the risk of adverse events and early mortality. Because ChD is associated with a high mortality [18], STE may be a relevant imaging technique to determine the prognosis in patients with CHF and VA.

LGE is an excellent imaging technique for detecting high arrhythmogenic risk patients and to identify those at the higher risk of SCD. However, GLS is also emerging as a potential tool for risk stratification in patients with ChD. Abnormalities in the inferolateral wall should be evaluated with STE in patients with ChD as a predictor for VA. The value of GLS as a predictor of cardiac mortality in ChD remains under investigation. Although LVEF is a strong predictor of death among cardiomyopathies, in CC and, GLS, the amount of fibrosis could have an equivalent role to predict adverse cardiac outcomes.

Finally, when CC is suspected, in both endemic and non-endemic geographical regions, a complete initial evaluation using STE may be advisable for optimal morphologic assessment.

## Figures and Tables

**Table 2.** **Systematic review equations. Research equations used to track articles in the different databases**.

DATABASE	RESEARCH EQUATION
PubMed	((cardiomyopathy, chagas [MeSH Terms]) OR (chagas disease [MeSH Terms])) AND (imaging, magnetic resonance [MeSH Terms]).
	(((cardiomyopathy, chagas [MeSH Terms]) OR (chagas disease [MeSH Terms]) OR (“chagas’ cardiomyopathy”.
	[MeSH Terms])) AND ((speckle tracking echocardiography) OR (speckle tracking strain) OR (myocardial strain)).
Embase	“Chagas disease OR chagas cardiomyopathy AND nuclear magnetic resonance OR magnetic resonance”.
	‘Chagas disease’ OR ‘Chagas cardiomyopathy’ AND ‘speckle tracking echocardiography’ OR ‘speckle tracking strain imaging’ OR ‘myocardial strain imaging’.
Cochrane	“Chagas cardiomyopathy in Keyword OR “echocardiography” in Title Abstract. Keyword OR speckle tracking imaging” in Title Abstract Keyword.
	Keyword: chagas cardiomyopathy AND title abstract key word “magnetic resonance imaging” OR title abstract key word “Chagas disease”.

**Table 3.** **Quality assessment of studies. JBI check lists for assessing the quality of studies classified by study type**.

Criteria cross sectional studies	BARROS [3]	MELLO [6]	TASSI [5]	NOYARABELO [7]	ROMANO [8]	MELENDEZ [9]
Were the criteria for inclusion in the sample clearly defined?	Y	Y	Y	Y	Y	Y
Were the study subjects and the setting described in detail?	Y	Y	Y	Y	Y	Y
Was the exposure measured in a valid and reliable way?	Y	Y	Y	Y	Y	Y
Were objective, standard criteria used for measurement of the condition?	Y	Y	Y	Y	Y	Y
Were confounding factors identified?	N	Y	N	U	Y	U
Were strategies to deal with confounding factors stated?	U	Y	Y	U	Y	N
Were the outcomes measured in a valid and reliable way?	Y	Y	Y	Y	Y	Y
Was appropriate statistical analysis used?	Y	Y	Y	Y	Y	Y
**total YES (n)**	6/8	8/8	7/8	6/8	8/8	6/8
**total YES (%)**	75%	100%	88%	75%	100%	75%
Criteria cohort studies				SENRA [10]	SANTOS JUNIOR [11]	
Were the two groups similar and recruited from the same population?				NA	Y	
Were the exposures measured similarly to assign people to both exposed and unexposed groups?				Y	Y	
Was the exposure measured in a valid and reliable way?				Y	Y	
Were confounding factors identified?				Y	Y	
Were strategies to deal with confounding factors stated?				Y	Y	
Were the groups/participants free of the outcome at the start of the study (or at the moment of exposure)?				Y	Y	
Were the outcomes measured in a valid and reliable way?				Y	Y	
Was the follow up time reported and sufficient to be long enough for outcomes to occur?				Y	Y	
Was follow up complete, and if not, were the reasons to loss to follow up described and explored?				Y	Y	
Were strategies to address incomplete follow up utilized?				U	NA	
Was appropriate statistical analysis used?				Y	Y	
**total YES (n)**				9/10	10/10	
**total YES (%)**				82%	100%	
Criteria case-control studies			VOLPE [12]	LIMA [13]	CIANCIULLI [14]	GOMES [15]
Were the groups comparable other than the presence of disease in cases or the absence of disease in controls?			Y	Y	Y	Y
Were cases and controls matched appropriately?			Y	Y	Y	U
Were the same criteria used for identification of cases and controls?			N	Y	Y	Y
Was exposure measured in a standard, valid and reliable way?			Y	Y	Y	Y
Was exposure measured in the same way for cases and controls?			Y	Y	Y	Y
Were confounding factors identified?			Y	N	Y	U
Were strategies to deal with confounding factors stated?			N	N	Y	N
Were outcomes assessed in a standard, valid and reliable way for cases and controls?			Y	Y	Y	Y
Was the exposure period of interest long enough to be meaningful?			Y	Y	U	Y
Was appropriate statistical analysis used?			Y	Y	Y	Y
**total YES (n)**			8/10	8/10	9/10	7/10
**total YES (%)**			80%	80%	90%	70%

Y, Yes; N, No; U, Unclear; NA, Not Applicable.

## Appendix

See Tables 2,3.
